# Near-death experience and quality of life: additional research

**DOI:** 10.1186/s13054-023-04465-y

**Published:** 2023-05-12

**Authors:** Bruce Greyson

**Affiliations:** Division of Perceptual Studies, Department of Psychiatry and Neurobehavioral Sciences, University of Virginia Health System, 210 10th Street NE, Charlottesville, VA 22902-4754 USA

Dear Editor,

I read with great interest the recent article by Rousseau et al. [1] documenting the incidence of near-death experiences (NDEs) in intensive care unit (ICU) patients, and noting that NDEs, contrary to expectation, were not significantly associated with quality of life one year later, as measured by the World Health Organization Quality of Life-Spirituality, Religiousness and Personal Beliefs (WHOQOL-SRPB). As the authors noted, NDEs are typically reported as life-transforming, but may be associated with distressing emotions [2].

The authors acknowledged some limitations that may have influenced the finding of no effect of NDEs on quality of life, such as the limited number of patients who reported NDEs. However, prior research has documented a similar lack of effect on quality of life following NDEs, using different measures. Olson and Dulaney [3], in a comparably small sample of elderly patients, found no difference in quality of life as measured by the Life Satisfaction Index-A between those who did and did not report NDEs. Greyson [4], sampling 126 individuals with NDEs, found no difference in quality of life as measured by the Satisfaction With Life Scale compared with those who did not have NDEs and those who had not come close to death.

These similar findings using different measures of quality of life and different populations support the finding of Rousseau et al. that the reported positive personality transformations following NDEs do not lead to improved quality of life. It is plausible that adjustment problems following NDES are sufficient to offset positive changes in attitudes, beliefs, and personality in influencing quality of life [4]. It has also been suggested by the facilitator of an NDE support group that, whereas most people assess their quality of life in comparison with an idealized earthly life, people who report NDEs may compare their current quality of life with memories of their perceived experience in a transcendental realm, which may lead them to diminish their ratings of their current quality of life [5]. Whatever the explanation, the lack of influence of NDE on subsequent quality of life appears to be a robust finding and not an artifact of limited sample size.

## Data Availability

No previously unpublished data were discussed in this Correspondence.

## Abbreviations

NDE: Near-death experience
WHOQOL-SRPB: World Health Organization Quality of Life-Spirituality, Religiousness and Personal Beliefs
